# Influenza Vaccination Coverage Among Pregnant Women — United States, 2016–17 Influenza Season

**DOI:** 10.15585/mmwr.mm6638a2

**Published:** 2017-09-29

**Authors:** Helen Ding, Carla L. Black, Sarah Ball, Rebecca V. Fink, Walter W. Williams, Amy Parker Fiebelkorn, Peng-Jun Lu, Katherine E. Kahn, Denise V. D’Angelo, Rebecca Devlin, Stacie M. Greby

**Affiliations:** ^1^Immunization Services Division, National Center for Immunization and Respiratory Diseases, CDC; ^2^Abt Associates, Inc., Cambridge, Massachusetts; ^3^Division of Reproductive Health, National Center for Chronic Disease Prevention and Health Promotion, CDC.

Pregnant women and their infants are at increased risk for severe influenza-associated illness (*1*), and since 2004, the Advisory Committee on Immunization Practices (ACIP) has recommended influenza vaccination for all women who are or might be pregnant during the influenza season, regardless of the trimester of the pregnancy (*2*). To assess influenza vaccination coverage among pregnant women during the 2016–17 influenza season, CDC analyzed data from an Internet panel survey conducted during March 28–April 7, 2017. Among 1,893 survey respondents pregnant at any time during October 2016–January 2017, 53.6% reported having received influenza vaccination before (16.2%) or during (37.4%) pregnancy, similar to coverage during the preceding four influenza seasons. Also similar to the preceding influenza season, 67.3% of women reported receiving a provider offer for influenza vaccination, 11.9% reported receiving a recommendation but no offer, and 20.7% reported receiving no recommendation; among these women, reported influenza vaccination coverage was 70.5%, 43.7%, and 14.8%, respectively. Among women who received a provider offer for vaccination, vaccination coverage differed by race/ethnicity, education, insurance type, and other sociodemographic factors. Use of evidence-based practices such as provider reminders and standing orders could reduce missed opportunities for vaccination and increase vaccination coverage among pregnant women.*

Since 2011, an Internet panel survey has been conducted for CDC by Abt Associates, Inc. (Cambridge, Massachusetts) at the beginning of each April to provide end-of-season estimates of influenza vaccination coverage among pregnant women and assess factors associated with vaccination. The Internet panel^†^ and survey methodology have been described previously (*3*). The 2016–17 survey was conducted during March 28–April 7, 2017, among women aged 18–49 years who reported being pregnant at any time since August 1, 2016. Among 10,734 women who entered the survey site, 2,399 were eligible and 2,319 completed the survey (a cooperation rate of 96.7%).^§^ Data were weighted to reflect the age, race/ethnicity, and geographic distribution of the total U.S. population of pregnant women. A woman was considered to be vaccinated for the 2016–17 season if she reported receiving vaccination before or during her most recent pregnancy since July 1, 2016. Analysis was limited to 1,893 women who reported being pregnant any time during the peak influenza vaccination period (October 2016–January 2017). A difference was noted as an increase or decrease when a ≥5 percentage-point difference occurred between any values being compared.^¶^

Influenza vaccination coverage among pregnant women in 2016–17 was similar to coverage during the previous four seasons (Figure). Among women pregnant during the 2016–17 influenza season, 53.6% reported receiving influenza vaccination before (16.2%) or during (37.4%) pregnancy since July 1, 2016 (Table 1). Coverage among women aged 18–24 years (41.7%) was lower than coverage among women aged 25–34 years (58.4%) and 35–49 years (58.5%). Coverage among Hispanic women (61.2%) was higher than that among non-Hispanic white (white) women (55.4%) and non-Hispanic black (black) women (42.3%); these differences were not observed during the 2015–16 season. Higher vaccination coverage was found among women with higher level of education, married women, women with private or military insurance, working women, women at or above poverty level, women with a high-risk condition, women with positive attitude toward vaccination effectiveness or safety, and women who were concerned about influenza infection, similar to the 2015–16 season.

**FIGURE:**
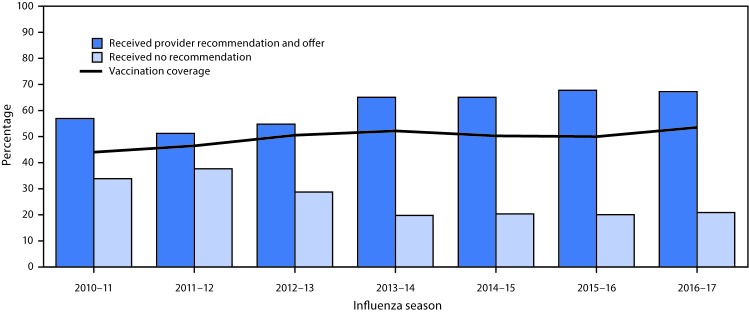
Prevalence of provider recommendation for and offer of influenza vaccination* and influenza vaccination coverage^†^ among women pregnant any time during October–January — Internet panel survey, United States, 2010–11 through 2016–17 influenza seasons * Among women who reported having at least one visit to a provider since July. ^†^ Vaccination coverage estimates for the 2012−13 through 2016−17 influenza seasons were based on vaccination given from July to mid-April; coverage estimates for the 2010−11 and 2011−12 influenza seasons were based on vaccination given from August to mid-April.

**TABLE 1:** Influenza vaccination coverage before and during pregnancy among women pregnant any time during October−January, by selected characteristics — Internet panel surveys, United States, 2016−17 and 2015−2016 influenza seasons

Characteristic	2015–16 influenza season			2016–17 influenza season			Percentage point difference in vaccination coverage 2016–17 to 2015–16
	Unweighted no.	Weighted %	Vaccinated, weighted %	Unweighted no.	Weighted %	Vaccinated, weighted %	
**Total**	**1,692**	**—**	**49.9**	**1,893**	**—**	**53.6**	**3.7**
Vaccinated before pregnancy	239	—	14.1	292	—	16.2	2.1
Vaccinated during pregnancy	605	—	35.8	750	—	37.4	1.6
**Age group (yrs)**							
18–24	417	28.9	49.4	464	28.6	41.7*	-7.7^†^
25–34	981	53.6	49.8	1,087	53.8	58.4	8.6^†^
35–49^§^	294	17.5	51.2	342	17.6	58.5	7.3^†^
**Race/Ethnicity**							
Hispanic	366	22.1	51.8	257	21.5	61.2*	9.3^†^
Black, non-Hispanic	277	19.8	49.4	262	20.8	42.3*	-7.1^†^
White, non-Hispanic^§^	898	50.4	49.0	1,200	50.2	55.4	6.4^†^
Other, non-Hispanic	151	7.7	52.1	174	7.5	51.7	-0.4
**Education**							
<College degree	872	53.1	46.5*	672	37.9	47.3*	0.8
College degree	642	36.8	52.6*	910	46.4	52.7*	0.1
>College degree^§^	178	10.2	58.2	311	15.7	71.7	13.6^†^
**Married**							
Yes^§^	1,044	59.8	53.5	1,386	70.2	56.7	3.2
No	648	40.2	44.6*	507	29.8	46.4*	1.8
**Insurance coverage**							
Any public	672	41.3	46.8*	568	32.9	47.6*	0.8
Private/Military only^§^	983	56.6	53.5	1,250	63.0	59.3	5.8^†^
No insurance	37	2.1	14.9*	75	4.1	14.6*	-0.3
**Working status^¶^**							
Yes^§^	950	56.1	53.9	1,239	65.4	57.1	3.2
No	742	43.9	44.9*	654	34.6	47.2*	2.3
Poverty status**							
At or above poverty^§^	1,312	76.4	52.0	1,688	88.2	55.1	3.2
Below poverty	377	23.6	43.1*	204	11.8	42.5*	-0.6
**High-risk condition^††^**							
Yes^§^	728	43.0	55.6	729	38.2	63.3	7.7^†^
No	964	57.0	45.7*	1,164	61.8	47.7*	2.0
**Number provider visits since July**							
None	10	0.6	–^§§^	69	4.3	6.1*	
1–5	326	19.6	39.5*	430	22.6	39.8*	0.3
6–10	706	41.5	50.0*	720	37.9	58.8	8.7^†^
>10^§^	650	38.3	55.7	674	35.2	62.7	7.0^†^
**Provider recommendation/offer^¶¶^**							
Recommended and offered^§^	1,133	67.6	63.4	1,238	67.3	70.5	7.1^†^
Recommended with no offer	218	12.5	37.5*	221	11.9	43.7*	6.2^†^
No recommendation	331	19.9	12.8*	363	20.7	14.8*	2.0
Attitude toward effectiveness of influenza vaccination***							
Positive^§^	1,313	77.9	61.8	1,473	77.8	65.8	4.0
Negative	379	22.1	8.0*	420	22.2	10.8*	2.8
**Attitude toward safety of influenza vaccination^†††^**							
Positive^§^	1,265	74.6	62.8	1,467	75.4	66.9	4.1
Negative	427	25.4	12.2*	426	24.6	12.9*	0.7
**Attitude toward influenza infection^§§§^**							
Concerned^§^	1,059	62.9	54.0	1,231	64.6	58.8	4.7
Not concerned	633	37.1	43.0*	662	35.4	44.3*	1.3

* ≥5 percentage-point difference compared with reference group.

^†^ ≥5 percentage-point difference from 2015–16 to 2016–17 influenza season.

^§^ Reference group for comparison within subgroups.

^¶^ Women who were employed for wages and self-employed were categorized as working; those who were out of work, homemakers, students, retired, or unable to work were categorized as not working.

** As determined by the U.S. Census Bureau (https://www.census.gov/data/tables/time-series/demo/income-poverty/historical-poverty-thresholds.html). For 2016–17 season, below poverty = a total of annual family income <$24,339 for a family of four with two minors as of 2016; for 2015–16 season, below poverty = total family income of <$24,036 for a family of four with two minors as of 2015.

^††^ Conditions associated with increased risk for serious medical complication from influenza, including chronic asthma, a lung condition other than asthma, a heart condition, diabetes, a kidney condition, a liver condition, obesity, or a weakened immune system caused by a chronic illness or by medicines taken for a chronic illness.

^§§^ Vaccination coverage estimates were suppressed because sample size was <30.

^¶¶^ Excluded women who had no provider visit since July 2016 (n = 69) for 2016−17 influenza season and women who had no provider visit since July 2016 (n = 10) for 2015−16 influenza season.

*** Created based on two questions regarding attitudes toward effectiveness of influenza vaccination: “Flu vaccine is somewhat/very effective in preventing flu”; and “Flu vaccine a pregnant woman received is somewhat/very effective in protecting her baby from the flu.” One point was given for each “yes” answer for either of the two questions. Respondents with a summary score of 1 or 2 were considered to have a “positive” attitude, and those with a summary score of 0 were considered to have a “negative” attitude.

^†††^ Created based on three questions regarding the safety of influenza vaccination: “Flu vaccination is somewhat/very/completely safe for most adult women”; “Flu vaccination is somewhat/very/completely safe for pregnant women”; and “Flu vaccination that a pregnant woman receives is somewhat/very/completely safe for her baby.” One point was given for each “yes” answer for any of the three questions. Respondents who had a summary score of 2 or 3 were considered to have a “positive” attitude, and those with a summary score of 1 or less were considered to have a “negative” attitude.

^§§§^ Created based on response to three questions regarding attitudes regarding influenza infection: “If a pregnant women gets the flu, it is somewhat/very likely to harm the baby”; “Flu infection during pregnancy is somewhat/very likely harm pregnant women”; and “Flu infection during pregnancy somewhat/very likely harm her baby.” One point was given for each “yes” answer for any of the three questions. Respondents who had a summary score of 2 or 3 were considered to be “concerned” and those with a summary score of 1 or less were considered to be “not concerned.”

The proportion of women who reported receiving a provider recommendation for and offer** of vaccination was 67.3% in the 2016–17 season, similar to that during the past four seasons (Figure). During both the 2015–16 and 2016–17 seasons, women who reported receiving both a provider recommendation for and offer of influenza vaccination had higher vaccination coverage (63.4% [2015–16] and 70.5% [2016–2017]) compared with women who reported receiving a provider recommendation but no offer^††^ (37.5% and 43.7%) and women who reported receiving no recommendation for vaccination^§§^ (12.8% and 14.8%) (Table 1); this pattern was observed among all age groups, racial/ethnic groups, levels of education, marital status, some level of insurance coverage, poverty status, number of health care visits, presence or absence of a high-risk condition, attitudes regarding efficacy and safety of influenza vaccine, and concern about influenza infection (Table 2). An increased number of provider visits since July 2016 was associated with both an increase in women’s report of receiving a provider recommendation and an increase in vaccination coverage estimates (65.7% [1–5 visits]; 70.9% [6–10 visits]; 72.1% [>10 visits]). Women in the following subgroups reported receiving a provider recommendation for and offer of vaccination less frequently than did women in the reference category of each stratum: aged 18–24 years, with a college degree, without medical insurance, without a high-risk condition other than pregnancy, with a negative attitude toward influenza vaccination effectiveness or safety, or not concerned about influenza infection (Table 2).

**TABLE 2:** Percentage of women receiving a provider recommendation/offer of influenza vaccination and self-reported influenza vaccination coverage, by provider recommendation and offer among women who visited a provider at least once since July 2016 and who were pregnant any time during October 2016–January 2017 — Internet Panel Survey, United States, 2016–17 influenza season

Characteristic	Provider recommendation for/offer of influenza vaccination				Vaccination coverage					
	Unweighted no.	Recommended, offered, weighted %	Recommended, no offer, weighted %	No recommendation, weighted %	Provider recommended, offered		Provider recommended, no offer		No recommendation	
					Unweighted no.	Weighted %	Unweighted no.	Weighted %	Unweighted no.	Weighted %
**Total**	**1,822**	**67.3**	**11.9**	**20.7**	**1,238**	**70.5**	**221**	**43.7**	**363**	**14.8**
**Age group (yrs)**										
18–24	408	61.0*	10.6	28.3*	249	65.2*	46	26.7*	113	14.8
25–34	1,074	69.1	12.6	18.3	746	72.6	136	49.6	192	14.5
35–49^†^	340	71.1	11.7	17.2	243	71.1	39	46.9	58	15.8
**Race/Ethnicity**										
Hispanic	254	70.1	9.1	20.8	181	75.8*	23	─^§^	50	21.5*
Black, non-Hispanic	216	64.4	12.3	23.3	137	64.9*	25	─^§^	54	9.8
White, non-Hispanic^†^	1,180	67.7	13.0	19.3	807	70.8	153	44.6	220	13.6
Other, non-Hispanic	172	64.1	12.2	23.7	113	65.7	20	─^§^	39	16.6
**Education**										
<College degree	660	67.6	10.2	22.2	443	62.0*	69	30.3*	148	13.3*
College degree	853	65.4*	12.3	22.3	573	73.6*	106	41.6*	174	15.0*
>College degree^†^	309	71.9	15.1	13.0	222	82.1	46	70.2	41	20.3
**Married**										
Yes^†^	1,330	68.4	12.9	18.7	920	73.1	172	51.1	238	15.3
No	492	65.0	9.6	25.4	318	64.3*	49	20.9*	125	14.0
**Insurance coverage**										
Private/Military only^†^	1,221	68.3	12.8	18.9	847	74.7	163	48.7	211	17.8
Any public	540	69.3	10.4	20.3	371	63.9*	53	31.8*	116	12.0*
No insurance	61	30.2*	9.8	60.0	20	─^§^	5	─^§^	36	6.2
**Working status^¶^**										
Yes^†^	1,176	68.0	12.3	19.7	803	74.8	152	49.8	221	17.4
No	646	66.2	11.3	22.5	435	62.7*	69	32.0*	142	10.8*
Poverty status**										
At or above poverty^†^	1,624	66.7	12.3	21.0	1,099	73.0	203	46.9	322	14.5
Below poverty	197	72.2*	8.9	18.9	138	54.1*	18	─^§^	41	17.6
**High-risk condition^††^**										
Yes^†^	724	75.1	10.3	14.6	546	75.3	74	48.5	104	14.1
No	1,098	62.2*	13.0	24.8	692	66.7*	147	41.3*	259	15.1
**Number of provider visits since July 2016**										
1–5	429	48.3*	13.5	38.3	217	65.7*	58	25.4*	154	12.2*
6–10	720	71.4	11.8	16.8	517	70.9	85	46.0*	118	16.1
>10^†^	673	75.2	11.1	13.7	504	72.1	78	55.3	91	17.8
**Attitude toward efficacy of influenza vaccination^§§^**										
Positive^†^	1,430	72.0	11.4	16.7	1,037	80.5	164	54.5	229	22.4
Negative	392	50.3*	14.1	35.7	201	17.9*	57	11.7*	134	1.9*
**Attitude toward safety of influenza vaccination^¶¶^**										
Positive^†^	1,421	73.8	11.6	14.6	1,047	80.4	169	56.3	205	22.6
Negative	401	47.0*	13.0	40.0	191	21.3*	52	8.1*	158	5.9*
Attitude toward influenza infection***										
Concerned^†^	1,182	70.1	11.5	18.4	839	74.5	139	50.7	204	17.7
Not concerned	640	62.3*	12.8	25.0	399	62.5*	82	32.4*	159	11.0*

* ≥5 percentage point difference compared with reference group.

^†^ Reference group for comparisons within subgroups.

^§^ Vaccination coverage estimates were suppressed because sample size was <30.

^¶^ Persons who were employed for wages and self-employed were categorized as working. Those who were out of work, homemakers, students, retired, or unable to work were categorized as not working.

** As determined by the U.S. Census Bureau (https://www.census.gov/data/tables/time-series/demo/income-poverty/historical-poverty-thresholds.html). For 2016–17 season, below poverty = a total of annual family income <$24,339 for a family of four with two minors as of 2016.

^††^ Conditions associated with increased risk for serious medical complication from influenza, including chronic asthma, a lung condition other than asthma, a heart condition, diabetes, a kidney condition, a liver condition, obesity, or a weakened immune system caused by a chronic illness or by medicines taken for a chronic illness.

^§§^ Created based on two questions regarding attitudes toward influenza vaccination: “Flu vaccine is somewhat/very effective in preventing flu”; and “Flu vaccine a pregnant women received is somewhat/very effective in protecting her baby from the flu.” 1 point was given for each “yes” answer for either of the two questions. Respondents with a summary score of 1 or 2 were defined to have a “positive” attitude, and those with a summary score of 0 were defined to have a “negative” attitude.

^¶¶^ Created based on three questions regarding the safety of influenza vaccination: “Flu vaccination is somewhat/very/completely safe for most adult women”; “Flu vaccination is somewhat/very/completely safe for pregnant women”; and “Flu vaccination that a pregnant women receives is somewhat/very/completely safe for her baby.” 1 point was given for each “yes” answer for any of the three questions. Respondents who had a summary score of 2 or 3 were defined to have a “positive” attitude, and those with a summary score of 1 or less were defined to have a “negative” attitude.

*** Created based on response to three questions regarding attitude toward influenza infection: “If a pregnant women gets the flu, it is somewhat/very likely to harm the baby”; “Flu infection during pregnancy somewhat/very likely harm pregnant women”; and “Flu infection during pregnancy somewhat/very likely harm her baby.” 1 point was given for each “yes” answer for any of the three questions. Respondents who had a summary score of 2 or 3 were defined as “Concerned” and those with a summary score of 1 or less were defined as “Not concerned.”

Vaccination coverage differed within some subgroups that reported similar proportions of receipt of a provider recommendation for and offer of vaccination. For example, although 68%–69% of insured women reported being offered vaccination, coverage was 74.7% among women with private or military insurance and 63.9% among women with public insurance. Differences in coverage among women who were offered vaccination were also observed between white and black women and women with more than a college degree and those with a college degree or less (Table 2). Among insured women who were offered vaccination, a higher proportion of publically insured women were younger (18–24 years), black, had less than a college degree, and lived below the poverty threshold compared with privately insured women.

Among the 221 (11.9%) women who reported that their provider recommended but did not offer vaccination, 114 (51.0%) received a referral^¶¶^ to go somewhere else to be vaccinated; 36.7% of the women receiving a referral were vaccinated, compared with only 12.5% of women who received a provider recommendation but no offer or referral.

## Discussion

Influenza vaccination coverage among pregnant women in 2016–17 was 53.6%, similar to coverage in the 2012–13 through 2015–16 influenza seasons. Similar to the past three seasons, 67.3% of pregnant women in 2016–17 reported receiving a provider recommendation for and offer of vaccination. Although the Standards for Adult Immunization Practice (*4*) support recommendation for and offer of influenza vaccination, the percentage of currently or recently pregnant women who reported receiving a provider recommendation and offer has not changed during the last four influenza seasons. This might be partly attributable to differences in perception among patients and providers of a recommendation for or offer of vaccination. In a recent survey of obstetric care providers conducted by the American College of Obstetricians and Gynecologists (ACOG), all surveyed providers reported that they recommend influenza vaccine to their pregnant patients; however, only 85% of patients surveyed at the same practices reported receiving a recommendation for vaccination, suggesting that although providers believe they are giving a recommendation for vaccination, the recommendation might not be communicated effectively (*5*).

Vaccination differences were seen by race/ethnicity, concerns about vaccination and influenza, insurance status, and number of provider visits. As has previously been observed, black women had lower vaccination coverage and Hispanic women had higher vaccination coverage compared with white women, despite similar percentages among each racial/ethnic group reporting a provider recommendation for and offer of vaccination (*3*). One study found that racial differences in vaccination coverage among pregnant women persisted after adjustment for a provider recommendation for or offer of influenza vaccination, insurance status, and demographic factors (*6*), and another study suggests that racial disparities might be caused by differences in sociocultural norms, misperception of effectiveness and safety of vaccination, and vaccination resistance and hesitancy (*7*), or could be modified or confounded by other factors such as age, education, or insurance status.

Although many women reported concerns about the safety or effectiveness of vaccination, these women were more likely to be vaccinated when there was a provider recommendation and offer compared with women with vaccination concerns who did not receive a vaccination recommendation from their provider, underscoring the need for providers to educate and counsel all pregnant patients. Although vaccination coverage increased with number of provider visits, 37% of women who had more than 10 visits were not vaccinated, indicating missed vaccination opportunities. Assessing vaccination status at every clinical encounter and providing an effective recommendation for and offer of vaccination can help ensure that more pregnant women receive influenza vaccine during pregnancy (*4*). ACOG has developed a toolkit to assist providers in integrating vaccination services and effective recommendations into their practice, including communication strategies and other resources.***

In this report, vaccination coverage was lower among pregnant women with public health insurance than among those with private or military insurance, at each level of provider recommendation for or offer of vaccination; frequency of provider recommendation or offer was similar for women with public and private or military insurance. This was also found among women with less than a college degree compared with women with more than a college degree. Lower vaccination coverage has been reported among pregnant women with public insurance (*8*) and women with lower levels of education (*3*).^†††^ Further work is needed to understand and address barriers to receipt of influenza vaccination by pregnant women covered by public insurance and with less than a college degree.

The findings in this report are subject to at least four limitations. First, a nonprobability sample that did not include women without Internet access was used in the analysis; therefore, results are not generalizable to all pregnant women in the United States. Second, vaccination status was self-reported and might be subject to recall bias or social desirability bias. Third, because the Internet panel survey is an opt-in survey, estimates might be biased if a woman's decision to join the internet panel or participate in this particular survey were related to receipt of vaccination. Vaccination coverage estimates from the Internet panel survey have been consistently 5–10 percentage points higher than estimates from the less timely probability-based National Health Interview Survey. However, both surveys have found similar stable trends with no increasing coverage.^§§§^ Strengths and limitations of the Internet panel survey compared with probability sampling surveys can be found elsewhere (*9*). Finally, the composite variables computed for attitudes toward influenza vaccination and infection were not validated.

Findings in this report support evidence that a provider’s recommendation for and offer of influenza vaccination to pregnant women is associated with receipt of vaccination. Women who were referred to another provider for vaccination were more likely to be vaccinated than women who did not receive an offer or referral. The Standards for Adult Immunization Practices call for all providers to strongly recommend needed vaccines and either administer vaccines or refer patients to a provider who can administer them (*4*). ACOG and Text4Baby^¶¶¶^ provide resources to ensure recommendations are provided effectively to help women receive influenza vaccination as early as possible during pregnancy. Vaccination coverage of pregnant women can be increased by a combination of 1) implementation of evidence-based practices (e.g., provider reminders and standing orders for vaccination) to ensure that influenza vaccination is recommended and offered at each visit before and during pregnancy or that the patient is referred to an influenza vaccine provider, and 2) clinical education about the risk for influenza infection and safety and benefit of influenza vaccination (*10*). Further work is needed to understand differences in vaccination coverage among women who were offered vaccination by a provider.

**Summary**

**What is already known about this topic?**

Pregnant women and infants are at increased risk for influenza-related complications and hospitalization. Vaccinating pregnant women can reduce their risk for influenza-related respiratory illness and reduce the risk for influenza in their infants aged <6 months. A provider recommendation for and offer of vaccination is associated with higher vaccination coverage among pregnant women.

**What is added by this report?**

Analysis of data from a 2017 Internet panel survey indicates that in the 2016–17 influenza season, 53.6% of pregnant women were vaccinated before or during pregnancy, similar to the 2015–16 season. Prevalence of provider recommendation for and offer of vaccination were similar to those in the last four influenza seasons. Most women who reported receiving both a provider recommendation for and offer of influenza vaccination had high vaccination coverage (70.5%), but this varied for those with public insurance (63.9%) and by other sociodemographic factors.

**What are the implications for public health practice?**

To improve protection from complications of influenza for mothers and infants, measures to improve vaccination coverage are needed. Implementing the Standards for Adult Immunization Practice, which recommend all health care providers assess, recommend, administer or refer, and document vaccinations, can help ensure pregnant women are fully vaccinated. Evidence-based practices such as provider reminders and standing orders can help implement these standards and reduce missed opportunities for vaccination.

## Footnotes

* Guide to Community Preventive Services: Vaccination; https://www.thecommunityguide.org/topic/vaccination.

† Additional information on the online survey and incentives for participants is available at https://www.surveysampling.com.

§ An opt-in Internet panel survey is a nonprobability sampling survey. The denominator for a response rate calculation cannot be determined because no sampling frame with a selection probability is involved at the recruitment stage. Instead, the survey cooperation rate is provided.

¶ Additional information on obstacles to inference in nonprobability samples is available at http://www.aapor.org/Education-Resources/Reports/Non-Probability-Sampling.aspx.

** “Recommendation and offer” is based on a “yes” response to the question “Since July 2016, during any of your visits to a doctor, nurse, or medical professional, did any of these medical professionals offer to give you a flu vaccination during a visit?”

†† “Recommendation but no offer” is based on a “yes” response to the question “Since July 2016, during any of your visits to a doctor, nurse, or other medical professional, did any of these medical professionals recommend that you get a flu vaccination or tell you that you needed a flu vaccination?” and a “no” response to the question “Since July 2016, during any of your visits to a doctor, nurse, or medical professional, did any of these medical professionals offer to give you a flu vaccination during a visit?”

§§ “No recommendation” is based on a “no” response to the questions “Since July 2016, during any of your visits to a doctor, nurse, or other medical professional, did any of these medical professionals recommend that you get a flu vaccination or tell you that you needed a flu vaccination?” and “Since July 2016, during any of your visits to a doctor, nurse, or medical professional, did any of these medical professionals offer to give you a flu vaccination during a visit?”

¶¶ Referral is defined based on a “yes” response to the question “Did any doctor, nurse, or medical professional suggest that you go someplace else to get the flu vaccination?”

*** http://immunizationforwomen.org/providers/resources/toolkits/immunization.php.

††† https://archive.cdph.ca.gov/HealthInfo/discond/Documents/MihaFactSheet.pdf.

§§§ https://www.healthypeople.gov/2020/data-search/Search-the-Data#objid=6362;.

¶¶¶ https://www.text4baby.org.
